# Role of PACAP in Female Fertility and Reproduction at Gonadal Level – Recent Advances

**DOI:** 10.3389/fendo.2012.00155

**Published:** 2012-12-11

**Authors:** Dora Reglodi, Andrea Tamas, Miklos Koppan, Donat Szogyi, Laura Welke

**Affiliations:** ^1^Department of Anatomy, Lendulet PACAP-Research Team of the University of Pécs and Hungarian Academy of SciencesPécs, Hungary; ^2^Department of Obstetrics and Gynaecology, University of PécsPécs, Hungary; ^3^Department of Anatomy, Ross University School of MedicineRoseau, Commonwealth of Dominica

**Keywords:** PACAP, ovary, oocyte, ovulation, luteinization

## Abstract

Pituitary adenylate cyclase activating polypeptide (PACAP) is a pleiotropic neuropeptide, first isolated from hypothalamic extracts, but later shown in peripheral organs, such as endocrine glands, gastrointestinal system, cardiovascular system, and reproductive organs. PACAP plays a role in fertility and reproduction. Numerous studies report on the gonadal regulatory effects of PACAP at hypothalamo-hypophyseal levels. However, the local effects of PACAP at gonadal levels are also important. The present review summarizes the effects of PACAP in the ovary. PACAP and its receptors are present in the ovary, and PACAP plays a role in germ cell migration, meiotic division, follicular development, and atresia. The autocrine-paracrine hormonal effects seem to play a regulatory role in ovulation, luteinization, and follicular atrophy. Altogether, PACAP belongs to the ovarian regulatory peptides.

## Introduction

Pituitary adenylate cyclase activating polypeptide (PACAP) was originally isolated from the hypothalamus, and named after its cAMP increasing effect in pituitary cells (Miyata et al., 1989; Arimura, 2007). PACAP belongs to the vasoactive intestinal peptide (VIP)/secretin/glucagon peptide family. It occurs in two amino acid forms: PACAP38 and PACAP27, the longer form is the predominant peptide in mammals. The receptors of PACAP are the VPAC1 and VPAC2 receptors, which bind VIP and PACAP with equal affinity and the specific PAC1 receptor, which binds PACAP selectively (Miyata et al., 1989; Sherwood et al., 2000; Arimura, 2007; Vaudry et al., 2009; Brubel et al., 2010). After the discovery of PACAP, numerous studies described the regulatory effects of PACAP at hypothalamo-pituitary levels, affecting the synthesis and release of several releasing and pituitary hormones. However, soon after its discovery, it was found that PACAP is, and its receptors are, expressed in the peripheral organs, and high levels of the peptide were found in the gonads (Arimura et al., 1991). This immediately drew attention to the peptide playing a central role in fertility and reproduction, not only at central hormonal, but also at local levels.

The last two decades in PACAP research have revealed that PACAP influences fertility and reproduction at several levels. The present mini-review aims to summarize findings on the effects of PACAP in the ovary and how PACAP influences female gonadal functions at the ovarian level.

## PACAP in Mammalian Follicular Development

Primordial germ cells (oogonia) migrate from the wall of the yolk sac to the ovaries during early mammalian development. Ovarian germ cells then undergo mitosis and develop further into primary oocytes. Developing oocytes are surrounded by epithelial cells, which build the ovarian follicles. At first, follicular epithelial cells are flattened and surround the primary oocytes entering the first meiotic division. They subsequently are arrested during fetal development until the female reaches sexual maturity. The first meiotic division is only completed during the follicular development before ovulation in each cycle, and the second meiotic division is completed only at the time of fertilization, resulting in the haploid gamete (Sadler, 2012).

A cohort of primordial follicles is activated at the beginning of the maturation process in cyclic manner. Follicular maturation involves changes in the oocyte, the follicular epithelial cells, and the surrounding ovarian stroma. Although significant inter-species differences exist in the follicular growth and its regulation, the major steps show basic similarities in mammalian species, and in most mammals, the vast majority of follicles undergo apoptosis similarly to humans (Matsuda et al., 2012). Therefore, we will focus on mammalian findings and only briefly mention other species. Early follicular development is independent from gonadotropins, but later follicular growth, ovulation, and luteinization are primarily regulated by the pituitary gonadotropins follicle-stimulating hormone (FSH) and luteinizing hormone (LH). However, their actions are also dependent on other peptidergic and non-peptidergic signaling pathways (Mayerhofer et al., 1997; Bodis et al., 2001; Matsuda et al., 2012). Accumulating evidence shows that PACAP is one of the regulatory peptides in the ovary.

### Actions of PACAP on primordial germ cells

There is evidence for the involvement of PACAP in the proliferation of primordial germ cells (Pesce et al., 1996). The authors showed PACAP immunoreactivity in gonadal ridges, mostly on the germ cell surface. Primordial germ cells intensively proliferate during their migration to the gonadal ridges and PACAP was found to stimulate this proliferation process. The peptide binds to the germ cells and also to gonadal somatic cells (Pesce et al., 1996). These observations show that PACAP plays an important role in reproductive functions from the earliest stage of differentiation and development.

### PACAP in the ovary

In rat ovarian cells, PACAP positive cells were found in the preovulatory and ovulatory periods (Gras et al., 1996), with PACAP and PACAP mRNA positivity in granulosa and cumulus cells. Positivity was also found in stroma cells and theca cells and even nerve fibers innervating the ovary showed immunopositivity. These observations suggest that PACAP is involved in a number of processes regulated via hormonal as well as neuronal activity during follicular development in the ovary (Gras et al., 1996). Another group published on the existence of PACAP in the rat ovary the same year (Scaldaferri et al., 1996).

Pituitary adenylate cyclase activating polypeptide expression in the corpus luteum of the rat was subsequently shown by Kotani et al. (1997, 1998). Park et al. (2001) showed that PACAP mRNA was only expressed in the granulosa but not theca cells. This expression has a stage-specific regulation by GnRH in rat granulosa cells. While treatment of preovulatory granulosa cells with GnRH agonist stimulates PACAP mRNA expression, GnRH agonist treatment alone has no effect but reduces the FSH-induced PACAP mRNA levels. GnRH antagonist on the other hand had opposite effects: it inhibited induced PACAP gene expression in preovulatory cells while stimulating expression in immature granulosa cells. These results show that GnRH plays an important role in the regulation of ovarian PACAP expression and that PACAP expression is differentially regulated during the ovarian cycle (Park et al., 2001). The LH and FSH control of the PACAP expression was also confirmed by Lee et al. (1999). The involvement of progesterone receptors have also been shown in LH-induced PACAP gene expression (Ko et al., 1999). In addition, the gonadotropin-dependent regulation of PACAP mRNA has been confirmed in bovine preovulatory follicles (Sayasith et al., 2007). PACAP was found to be expressed in granulosa cells and its expression was found to be dependent on protein kinase A. A recent study has shown that PACAP mRNA expression gradually increases in pregnancy in the corpus luteum, suggesting its involvement in the maintenance of mid-term and late pregnancy (Zhao et al., 2010).

Transient expression of PACAP has also been observed in the mouse ovary within granulosa cells of preovulatory follicles after hCG treatment (Barberi et al., 2007). The contemporary induction of PACAP in preovulatory follicles suggests an important role for PACAP around the time of ovulation (Barberi et al., 2007). In human granulosa cells, it has been found that FSH and LH promote PACAP expression (Morelli et al., 2008). PACAP is synthesized as a preprohormone and is processed by prohormone convertases. It has been shown that in both testis and ovary, prohormone convertase 4 is the enzyme responsible for PACAP processing, supported by the finding that prohormone convertase 4 deficient mice expressed no PACAP in their ovaries (Li et al., 2000).

### Expression of PACAP receptors in the ovary

Earlier studies examining binding sites for PACAP have already identified specific binding sites in the ovary (Gottschall et al., 1990). Scaldaferri et al. (1996) identified isoforms of the PAC1 receptor in the rat ovary. Later, PAC1 receptors were shown in the rat corpus luteum (Kotani et al., 1997, 1998). Park et al. (2000) have demonstrated stage-specific expression of PACAP receptors in the rat ovary. Northern blot analysis shows that the ovarian transcript of the PAC1 receptor appears at day three in the rat ovary, followed by a gradual increase later in development. There is a marked increase at puberty, at the age of 21 days, when compared to an age of 15 days, when only non-growing small follicles are present. *In situ* hybridization has revealed that PACAP receptor mRNA is present mainly in the granulosa cells of the large preantral follicles, while atretic follicles and mural granulosa cells are devoid of the receptor. The authors have also demonstrated that the dominant splice variant of the PAC1 receptor was the short variant in the ovary (Park et al., 2000). Gonadotropin stimulation has been shown to induce PACAP receptor mRNA expression in the granulosa cells of the preovulatory follicles (Ko and Park-Sarge, 2000; Park et al., 2000). Pregnant mare serum gonadotropin (PMSG), on the other hand, causes reduction of PACAP receptor gene expression. PMSG induces multiple follicular growth to the preovulatory stage. These observations suggest an involvement of PACAP in the follicular growth, and in ovulation, in a stage- and time-dependent manner. The observation that PACAP receptor expression is restricted to granulosa cells of the growing follicles at the time of puberty and to granulosa cells of preovulatory follicles after gonadotropin treatment indicates that PACAP may act in a limited time-window in the ovary. Progesterone receptors have been shown to be involved in the induced PAC1 receptor expression (Ko and Park-Sarge, 2000).

A more detailed analysis of the rat ovarian PACAP receptor expression has confirmed the expression of PAC1 receptors in the granulosa cells and, furthermore, the presence of VPAC2 receptors in these cells (Vaccari et al., 2006). Theca cells do not express PAC1 receptors, only VPAC1 and 2 receptors. Fully developed oocytes only express the PAC1 receptor. hCG stimulation has been found to induce PAC1 receptor expression in granulosa and VPAC2 receptor expression in theca cells. The VPAC receptor expression has been found to have a lower expression level than the PAC1 receptor. This study has also confirmed the previous findings of Park et al. (2000) describing receptor expression at 3 days after birth with a marked increase before puberty. In addition, they found that VPAC1 receptors decreased with age and VPAC2 receptors remained constant. Immunohistochemical analysis revealed the presence of VPAC1 receptors in association with stromal blood vessels in the vicinity of the follicles, especially at the entrance site of the ovarian arteries into the medulla. The expression of the VPAC2 receptors was more ubiquitous in the ovary. Denuded oocytes express only the PAC1 receptor, which could not be detected in Met-I and II phases in oocytes matured *in vivo*. Gras et al. (2000) also described PAC1 and VPAC2 receptors in preovulatory follicles. All three types of the PACAP receptors have been identified in human granulosa cells, although the main receptor types in these cells seem to be the VPAC receptors (Morelli et al., 2008).

All three receptors have also been identified in the mouse (Barberi et al., 2007). PAC1 receptors could be stimulated by hCG, VPAC2 only mildly stimulated, and VPAC1 downregulated. The authors suggested that the contemporary induction of PACAP and PAC1 receptors by hCG in granulosa cells of preovulatory follicles indicates an important role for PACAP around the time of ovulation (Barberi et al., 2007).

### Actions of PACAP in the ovary

#### Follicular development, hormone production, and ovulation

In all mammalian species, the early stages of folliculogenesis, including the initiation of primordial follicle growth, are independent from gonadotropins (Latini et al., 2010). Fine balances between inhibitory and stimulatory signals regulate follicular recruitment. PACAP has been shown to inhibit primordial to primary follicle transition (Latini et al., 2010); PACAP did not influence granulosa cell viability at these stages but inhibited proliferation. PACAP also inhibited the growth of preantral follicles. These observations indicate an important role of PACAP in follicular recruitment (Latini et al., 2010). On the other hand, at later stages, PACAP can prime immature follicles (Gras et al., 2005). A 12-h PACAP priming stimulates FSH-induced estradiol production, which is an important step in cyclic recruitment, when a cohort of antral follicles escapes apoptosis and reaches the preovulatory stage (Gras et al., 2005). These results show a fine balance between factors, including PACAP, that play a role in follicular recruitment. Other studies have also shown that PACAP plays an important role in preantral follicular growth and differentiation (Cecconi et al., 2004). In the mouse ovary, PACAP or VIP alone did not affect follicular growth, but they both inhibited it when added to FSH-stimulated follicles. Both peptides caused a dose-dependent inhibition of follicle growth, antrum formation, granulosa cell proliferation, and estradiol production (Cecconi et al., 2004).

Studies on the endocrine effects of PACAP revealed that PACAP can stimulate cAMP accumulation and steroidogenesis in the rat ovary (Zhong and Kasson, 1994; Heindel et al., 1996; Vaccari et al., 2006). Granulosa cell cultures responded to PACAP treatment with increased production of estrogen, progesterone, and 20alpha-dihydroprogesterone. This effect was dose-dependent and more potent than similar effects of VIP and GHRH (Zhong and Kasson, 1994). The peptide was also able to augment FSH-induced progesterone and 20alpha-dihydroprogesterone accumulation, and in high doses that of estrogen. The PACAP-stimulated progesterone accumulation was minimal in the absence of androstendion and dramatically augmented in the presence of the androgen. This shows that androgens are required for the PACAP to stimulate steroid production and indicate that PACAP might play a role in modulating the production of steroids in the ovary (Zhong and Kasson, 1994). The effects of PACAP depend on the presence of LH. In cultured luteal cells of the rat, PACAP alone stimulates progesterone, 20alpha hydroxyl-4-pregnene-3-one along with cAMP accumulation (Usuki and Kotani, 2001, 2002). However, when LH is present, PACAP in high concentrations inhibits the LH-induced cAMP accumulation and progesterone production, while it enhances the LH-induced stimulation of 20alpha hydroxyl-4-pregnene-3-one production (Kotani et al., 1998). This decreases the ratio of progesterone to 20alpha hydroxyl-4-pregnene-3-one in LH-stimulated cells, which suggests an involvement in luteolysis. This is supported by the finding that PACAP suppresses increases in LH receptors in luteal cells (Usuki and Kotani, 2001). Gras et al. (2000) showed that PACAP can stimulate progesterone production and cAMP synthesis, and the involvement of the PKA pathway. These data together provide evidence for the involvement of PACAP as an autocrine-paracrine regulator of ovarian hormone production, especially in preovulatory follicles (Gras et al., 2000).

Interestingly, one intracerebroventricular PACAP treatment was shown to inhibit ovulation in adult animals (Koves et al., 1998). Injecting PACAP before the critical period of the proestrous stage, blocks the ovulation and prevents the proestrous LH surge (Koves et al., 1996). Furthermore, neonatal administration of PACAP delays onset of puberty in female animals (Szabo et al., 2002).

The effects of PACAP on ovarian steroidogenesis have been confirmed in human studies as well (Apa et al., 1997), where PACAP was found to stimulate progesterone synthesis without synergistic effects with hCG. This was tested in corpora lutea obtained from non-pregnant women undergoing hysterectomy.

PLC was activated by PACAP in granulosa but not theca cells (Vaccari et al., 2006). Plasminogen activators are known to be involved also in ovulation. PACAP has been shown to increase the tissue-type plasminogen activator and to decrease the urokinase-type one (Apa et al., 2002). VIP did not show this effect in granulosa cells, but when entire follicles were exposed to the peptides, both VIP and PACAP exerted similar stimulatory effects on tissue-type plasminogen activator levels, supporting the observations of different PACAP/VIP receptors being expressed in different ovarian compartments (Apa et al., 2002).

#### Apoptosis

In addition to gonadotropins, factors secreted from granulosa cells, including steroids, growth factors, and cytokines, are essential for granulosa cell survival and follicular growth (Matsuda et al., 2012). Granulosa cells are the initial population to undergo apoptosis in atretic follicles, indicating their role in the initiation of follicular atresia (Matsuda et al., 2012). Besides estradiol, as the main ovarian steroid influencing follicular apoptosis, several other factors play an important role. Among those, insulin-like growth factor, epidermal growth factor, fibroblast-like growth factor, and interleukin-1 beta have prosurvival effects, while the Fas ligand system and the tumor necrosis alpha are proapoptotic. Pro- and antiapoptotic members of the mitochondrial Bcl family have also been described to play an important role (Matsuda et al., 2012). A few papers suggest that PACAP is also one of the factors influencing granulosa cell death and survival.

One of the main effects of PACAP is its cell survival-promoting effects, which was first described in neuronal cells, and thus, PACAP was designated as an important neuroprotective peptide (Vaudry et al., 2002; Somogyvari-Vigh and Reglodi, 2004). However, numerous studies have revealed that the antiapoptotic effects of PACAP are not restricted to neuronal cells, but can be shown in several other cell types, such as lymphocytes (Delgado and Ganea, 2000), prostate cancer cells (Gutierrez-Canas et al., 2003), endothelial cells (Racz et al., 2007), retinal pigment epithelial cells (Fabian et al., 2012), and even in the invertebrate mollusks (Pirger et al., 2008). Therefore, PACAP can be considered as a general cytoprotective peptide. VIP, structurally the closest peptide to PACAP has been described to have antiapoptotic effects in the ovary (Flaws et al., 1995).

Based on these data, it is expected that PACAP can also exert antiapoptotic effects in ovarian cells. Indeed, several studies have shown this in granulosa cells. In isolated granulosa cells, apoptosis can be induced by serum-free culturing. In accordance with the generally known antiapoptotic role of PACAP, the peptide could reduce the granulosa cell apoptosis in a dose-dependent manner, an effect that could be blocked by the PACAP antagonist PACAP6-38 (Vaccari et al., 2006). These results are in accordance with earlier observations showing that PACAP could block the apoptotic cascade in granulosa cells (Lee et al., 1999) and that the LH suppression of follicle apoptosis was partially blocked by PACAP 6–38. The antiapoptotic effect of PACAP was also confirmed in human granulosa-luteal cells (Morelli et al., 2008). Both PACAP and VIP could reverse the decrease in procaspase-3 induced by the serum withdrawal. However, it was found that spontaneous apoptosis was not influenced by PACAP in another study (Gras et al., 2005).

Expression of PACAP in both benign and malignant ovarian tumors has been shown (Odum and Fahrenkrug, 1998). Higher levels were found in cancers than benign tumors. The authors suggest a trophic function of PACAP in ovarian cells (Odum and Fahrenkrug, 1998). Interestingly, PACAP does not influence the cisplatin-induced toxicity in proliferating ovary cells of the CHO ovarian cell line (Aubert et al., 2008). The same treatment, however, does prevent toxicity and related apoptosis in cerebellar granule cells. This dissociation in the antiapoptotic behavior of PACAP could be of high clinical importance: PACAP can decrease the cisplatin-induced neurotoxic side effects while leaving the antitumor therapeutic effect intact (Aubert et al., 2008).

#### Effects in oocytes

Fully developed oocytes express PAC1 receptor and the addition of nanomolar concentrations of PACAP induces calcium release. However, this answer could not be observed in Met-I and II phase oocytes, supporting that finding that receptors are expressed stage-specifically (Vaccari et al., 2006).

In *Xenopus* oocytes, PACAP has been described to modulate membrane potential by eliciting hyperpolarization-activated chloride current, thereby affecting oocyte physiology (Kato et al., 1997).

Very interesting results have been described by Apa et al. (1997) supporting both a direct and indirect effect of PACAP on oocyte maturation. Mammalian oocytes are known to arrest in the first meiotic division, which is resumed at the time of the preovulatory LH surge. The inhibition of oocyte maturation and its relief is mediated by gonadotropins in conjunct with several other factors, while only a few are known to act directly on oocytes. The authors described that PACAP accelerated meiotic maturation in follicle- and cumulus-enclosed oocytes while inhibiting meiotic maturation in denuded oocytes (Apa et al., 1997). This result was not due to a direct cytotoxic effect because the inhibition on oocyte maturation was reversible when PACAP was removed from the medium. This difference in PACAP action on enclosed and denuded oocytes support the stage-dependent regulatory effects of PACAP. Other studies have also confirmed the effect of PACAP on the meiotic processes. In the mouse ovary, Cecconi et al. (2004) found that PACAP severely impaired meiotic maturation in oocytes isolated from the follicles.

Recently, mass spectrometric and radioimmunoassay analysis have shown that PACAP is present in human follicular fluid obtained from patients undergoing hyperstimulation treatment (Brubel et al., 2011; Koppan et al., 2012). PACAP could be identified in all human samples examined. Correlation was found between retrieved oocytes and PACAP levels in the follicular fluid drawing the attention to PACAP as an important factor in the medium of the developing oocyte and its possible future use as a biomarker in women with fertility problems (Koppan et al., 2012).

## Evolutionary Perspectives

Recent reports point to the important roles of PACAP in fish reproduction, pointing to the fact that the effects of PACAP on the hypothalamo-pituitary axis, as well as in the gonads, are conserved and biologically ancient functions (Levy and Degani, 2011, 2012). In fish, stage-specific expression of PACAP has also been revealed. For example, higher PACAP expression can be found in female blue gourami, with oocytes in the final maturation stage, than in vitellogenic individuals. Also, higher expression was found in mature males that are not reproductively active than in nest builders and juveniles (Levy and Degani, 2012). PACAP is thus, differentially expressed in females and males. PACAP acts in close association with GnRH in fish reproduction and the expression and actions of PACAP also depend on environmental factors, such as temperature (Levy et al., 2011; Levy and Degani, 2012). It has been proposed that PACAP is involved in the final oocyte maturation in females and in males it is mainly associated with sexual behavior (Levy et al., 2010). In the zebrafish, a new type of PACAP has been described in the ovary and its role as an ovarian factor mediating gonadotropin action have been suggested (Wang et al., 2003).

## Role of Endogenous PACAP

Given the important functions of PACAP in reproduction outlined above, it would be expected that the lack of endogenous PACAP have major effects on fertility and reproduction. Indeed, PACAP deficient mice have been shown to have lower reproductive rates in several studies (Shintani et al., 2002; Sherwood et al., 2007; Isaac and Sherwood, 2008). To study the exact mechanism, a few studies examined the possible background for this lower reproductive rate. Interestingly, Isaac and Sherwood (2008) found that these mice had normal puberty onset, estrous cycle, and seminal plugs when paired with males. However, the birth rate after mating was only 20% when compared to the 100% in wild type mice. The authors found no defect in ovulation, ovarian histology, or fertilization, but they found major defects in implantation and associated hormone levels (Isaac and Sherwood, 2008). Similarly, low birth rates were found by Shintani et al. (2002), but they also found reduced mating and maternal behaviors. It is possible that in the ovary, compensatory mechanisms counteract the lack of PACAP and thus, no major alterations can be detected under normal circumstances. However, more studies are necessary to elucidate the exact role of endogenous PACAP in the ovary. Furthermore, in several other organs, including intestine, kidney, retina, and central nervous system, PACAP deficient mice do not exhibit major alterations unchallenged conditions (Reglodi et al., 2012). However, in case of stressors or under pathological conditions, PACAP deficient mice react with increased vulnerability and the observed deficits have been shown to be more severe in mice lacking endogenous PACAP (Reglodi et al., 2012). Based on these observations, it is possible that endogenous PACAP has ovarian functions not compensated under challenged or pathological conditions. Further studies are required to shed light on this possibility.

## Concluding Remarks

The present review summarized findings on the presence and effects of PACAP in the ovary. It has to be emphasized that this is only one role in the plethora of actions that PACAP plays in fertility and reproduction and that the detailed discussion of these is beyond the scope of the present review. Briefly, PACAP, at the hypothalamic level, influences receptive behavior in female rodents, in association with GnRH and steroids (Apostolakis et al., 2004, 2005), and plays an important modulatory role in pituitary hormone production. The role of PACAP in the hypothalamo-pituitary-gonadal axis has been reviewed several times previously (Rawlings and Hezareh, 1996; Nussdorfer and Malendowicz, 1998; Sherwood et al., 2000; Koves et al., 2003; Vaudry et al., 2006; Counis et al., 2007).

Pituitary adenylate cyclase activating polypeptide also plays a role in the muscle contraction of the vaginal wall as well as that of the uterus and uterine tube (Steenstrup et al., 1995; Ziessen et al., 2002), and even decreased immunoreactivity has been shown in premenopausal and postmenopausal women in the vaginal wall (Hong et al., 2008). The PACAPergic innervation of the female genital tract has also been shown and has been associated with nerves originating from the paracervical ganglia (Fahrenkrug and Hannibal, 1996; Fahrenkrug et al., 1996).

Effects of PACAP have also been shown in the placenta where PACAP and its receptors are present (Scaldaferri et al., 2000; Koh et al., 2005; Brubel et al., 2010), where the peptide influences blood flow in the utero-placental unit (Steenstrup et al., 1996) and influences survival of trophoblast cells (Boronkai et al., 2009). The serum level of PACAP increases in the third trimester of pregnancy in healthy pregnant women and it markedly decreases during delivery, reaching prebirth levels 3 days after delivery (Reglodi et al., 2010). High concentrations of PACAP have been shown in human and animal milk (Borzsei et al., 2009; Csanaky et al., 2012), which suggests that the peptide is also an important nutritional source for the newborn. However, its exact function in breastfeeding has not been identified yet.

In summary, PACAP is one of the peptides regulating germ cell development in the ovary and it has several other regulatory functions in reproduction. PACAP influences ovarian hormone production, affects meiosis, and is an important local regulator of follicular development. The exact physiological role and its possible clinical importance still awaits further investigation, but the current evidence points to a possible clinical diagnostic or therapeutic use of the peptide in both human and veterinary fertility.

## Conflict of Interest Statement

The authors declare that the research was conducted in the absence of any commercial or financial relationships that could be construed as a potential conflict of interest.
